# Holistic admission review in student nurse selection: A concept analysis

**DOI:** 10.4102/curationis.v49i1.2860

**Published:** 2026-06-20

**Authors:** Christel Joubert, Charlene Downing

**Affiliations:** 1 Department of Nursing, Faculty of Health Sciences, University of Johannesburg, Johannesburg, South Africa

**Keywords:** holistic admission review, holistic selection, student nurse selection, student nurse, concept analysis

## Abstract

**Background:**

Workforce diversity reduces health care disparities and enhances patient-centred care. Holistic admission review (HAR) in student nurse selection has been proposed to increase diversity in nursing. Nursing has been slow in the uptake of HAR. Additionally, South Africa nursing programme admissions primarily emphasise academic metrics. The focus on academic measures leads to the exclusion of academically successful, diverse and competent candidates.

**Objectives:**

This study sought to analyse the concept of HAR in the context of nursing education, addressing the question of the conceptual meaning of HAR in this context.

**Method:**

Walker and Avant’s eight-step concept analysis framework was implemented to establish a clear understanding of HAR. A scoping review, using Preferred Reporting Items for Systematic reviews and Meta-Analyses guidelines, was conducted across 10 databases and institutional repositories. Sources were screened using Covidence, and data extraction followed the conceptual analysis framework.

**Results:**

Out of 8558 screened sources, 84 were included in the analysis. Holistic admission review requires policies to address the use of a diverse selection committee using a standardised rubric to assess both cognitive and non-cognitive attributes.

**Conclusion:**

Holistic admission review is triggered by a lack of diversity and bias in selection processes. The HAR framework consists of four phases and ultimately fosters an inclusive student body equipped to deliver high-quality patient care. Holistic admission review enhances student retention, empowers future leaders and strengthens the nursing workforce.

**Contribution:**

This concept analysis will refine the research questions for subsequent phases, including exploring HAR’s meaning and developing a nursing education model for equitable student selection in South Africa.

## Introduction

### What is known?

Holistic admission review (HAR) has been used with success to select undergraduate students in multi-disciplinary fields. However, the meaning of HAR and how it should be implemented in nursing education are unclear.

### What is new?

A theoretical definition of HAR in nursing was formulated.

The results of the concept analysis of HAR will be used to describe a model to facilitate HAR within the context of nursing education.

### Background

The quality of nursing care and the values associated with nursing have been a point of deliberation in recent years (Scammell et al. 2017:218). Moreover, students’ values influence the care they provide within a specific clinical environment (Callwood et al. 2020:1). There is little doubt that individuals with a certain set of values would be more likely to provide greater care and higher quality nursing (McNeill et al. 2018:68). This has prompted the need to include desirable values-based attributes to underpin the selection of caring and compassionate nurses to the profession (Waugh et al. 2014:1195). One strategy included adding previous healthcare experience as an entry requirement (Capponi & Brown 2021:1028; Crawford et al. 2021:2507). Subsequently, holistic admission processes have been developed and adopted by nursing, broadening admission criteria beyond academic metrics (Morrow 2021:257).

Holistic admission can be defined as a flexible, individualised way of assessing an applicant’s capabilities for success, where balanced consideration is given to experiences and attributes as well as academic metrics (Curtis et al. 2015:2; Morrow 2021:257). Holistic admission processes are guided by the institutional mission, norms and objectives (Holmes & Bear 2022:21). These entail non-academic criteria (Capponi & Brown 2021:1034) as well as metrics such as grades and test scores (Jung et al. 2021:60; Lewis et al. 2021:714; Morrow 2021:256; Thompson & Sonke 2021:1086). These attributes are vital to selecting skilled individuals able to care for patients from diverse backgrounds (Jung et al. 2021:360). Using a holistic admission process is ultimately an important step in creating a workforce that reflects the demographics of the population seeking care (Lewis et al. 2021:719). The demographics relate to gender, race, nationality and language. This diversity also improves the outcome of the care provided (Jung et al. 2021:360). It stands to reason that should those who are providing care inherently display the same values as the population seeking the medical care, the quality of nursing care would be enhanced as trust would be increased by being like-minded. As it has been accepted that patient care and outcomes will improve with professional diversity (Morrow 2021:257), the use of a holistic admission process has been suggested. A holistic admission process in health care has been described in the literature for more than 20 years (Lewis et al. 2021:714). However, nursing has lagged behind other healthcare professions in implementing the holistic admission process (Jung et al. 2021:360). As a nurse and nurse educator in South Africa who has participated in the selection process as a candidate and as part of the selection committee, HAR has not been implemented yet. The value of HAR within the nursing profession, as well as the impact it can have on the quality of nursing care, is noted by the researcher. It is anticipated that, with organisational support, the process will start to move forward, as it has been adopted by some American universities (Lewis et al. 2021:715; Morrow 2021:256) and nursing schools (Jung et al. 2021:360). There is currently limited published literature on how this process is being implemented in nursing and the success of student outcomes (Morrow 2021:258).

## Research methods and design

This concept analysis entailed the first phase of a doctoral thesis and implemented the concept analysis process as described by Walker and Avant (2019:1701). Conceptual meaning of HAR is required, as in nursing, many concepts are abstract (Chinn, Kramer & Sitzman 2022:142).

### Study design

The study implemented the eight steps of concept analysis as outlined by Walker and Avant (2019:1701). These steps entail selecting the concept, determining the aims or purpose of the analysis, identifying all discernible uses of the concept, establishing defining attributes, identifying a model case, identifying other cases, identifying antecedents and consequences and finally, defining empirical referents (Nuopponen 2010:9). The steps allow for a systematic and structured approach to clarify and define the concept of HAR, which is essential for communication and application of the concept in nursing (Walker & Avant 2019:167).

### Data collection and analysis

A comprehensive search of 10 databases for studies published between 2013 and 2024 was conducted. The decision on which databases, institutional repositories (IR), as well as the timeline, was made by the researcher and study supervisor based on the merit of recent and applicable sources that would contribute to the body of knowledge. The selected databases included Ebscohost, Cinahl, Sabinet (SAe Publications), Web of Science, Proquest, Embase, JSTOR, Medline, Pubmed and Joanna Briggs Institute (JBI). The database search was conducted from 13 March 2024 to 13 August 2024. The IR accessed were determined by selecting the top five universities with nursing faculties, both nationally and internationally, in order to access valid and reliable nursing research. The researcher was able to access the following international universities’ IRs: Stanford, Massachusetts Institute of Technology and Harvard. The following national universities’ IRs were accessed: University of Cape Town, Stellenbosch and University of the Witwatersrand (WITS). The following university repositories allowed for viewing, but not for export: Oxford, Cambridge, KwaZulu-Natal and the University of Pretoria. This challenge was addressed by making use of the ProQuest database to export the relevant articles that were viewed on the repositories.

Inclusion criteria for the review were: Source must be written in English or Afrikaans, and source must entail steps, benefits or consequences on holistic admission selection of first-year or undergraduate students. Exclusion criteria were sources written in languages other than English and Afrikaans, sources older than 2013, selection processes of graduate or postgraduate students and all duplicates. These inclusion criteria describe critical or essential characteristics and features that distinguish the concept as a recognisable entity and that differentiate this entity from other related concepts (Chinn et al. 2022:144). The focus of the research is on HAR in the context of undergraduate student selection and thus informed the inclusion and exclusion criteria. In order to establish the search strings to be used, the researcher made use of Medical Subject Headings (MeSH) and had consensus discussions with the study supervisor. Ten search strings were established: HAR, holistic selection, holistic admission to faculty, selection and student nurse, selection and learner nurse, selection criteria and student nurse or learner nurse, admission criteria and student nurse or learner nurse, selection requirements and student nurse or learner nurse, selection criteria and graduate and lastly holistic admission. The researcher used an online app named Covidence to assist with rigour. The app excluded a total of 4887 duplicates. A total of 8558 sources were screened during the title and abstract screening process. A second researcher also conducted the title and abstract screening via the Covidence app. The screening was done independently, and the app ensured that it was done without the researchers knowing the vote, which contributed to the rigour and trustworthiness. All conflicts were then resolved by the researcher and the study supervisor. A total of 216 sources progressed to full-text review. The same review process was followed as per the title and abstract review. Eighty-four sources were then included as part of the extraction and analysis process.

## Results

### Selecting a concept

The concept selected for the analysis was HAR in student nurse selection. The selected concept, HAR, is widely used within selection processes across disciplines. However, limited data are available on the concept within nursing (Glazer et al. 2016a:306).

### Determining the purpose of the concept analysis

The aim of the concept analysis was to clarify the meaning of HAR by determining the defining attributes, meaning and how it may be a bridge to related work in nursing practice theory (Walker & Avant 2019:171). From this analysis, the antecedents, process and outcome of HAR were determined. The theoretical definition was developed in order to add value to the understanding of selection processes in nursing (Chinn et al. 2022:143).

### Identifying the scope of the concept use

Uses of the concept were determined using dictionaries, thesauruses and available literature (Walker & Avant 2019:172). The researcher conducted a thorough search of the literature to determine the extent of information available on HAR. The search strategy informed the inclusion and exclusion criteria. The researcher used a web-based application known as Covidence to assist with the review process. The systematic search using computerised databases increased the likelihood that sources were representative of the total population, which enhanced the credibility (Rodgers 1989:333). By sampling databases from various disciplines, it allowed for the exploration of information regarding the concept across a broad field of inquiry (Rodgers 1989:333). Search strings were developed by making use of MeSH as well as consensus discussions with the study supervisor.

**FIGURE 1:**
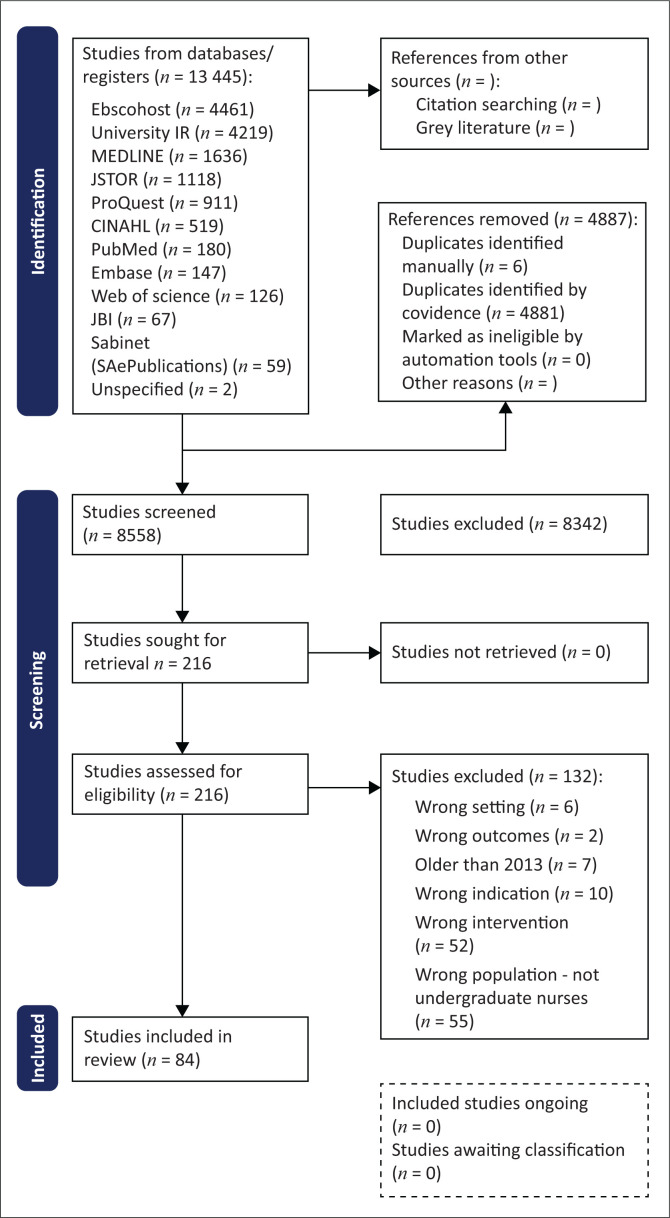
Preferred Reporting Items for Systematic reviews and Meta-Analyses for concept analysis of holistic admission review.

With the use of Covidence, of the 216 sources, 132 were excluded and 84 studies were used for data extraction (see Figure 1).

Covidence was also used during the data extraction period. The researcher was able to design a data extraction table with the following headings addressing concept analysis: Title of the article, author, date of publication, country of publication, antecedent, defining attributes, consequences and empirical referents. The researcher read and re-read the articles to complete the extraction table and allow the researcher to immerse in the data, which contributed to the trustworthiness of the extraction. The researcher read and re-read all the results in the extraction table to identify re-occurring themes for antecedents, defining attributes, consequences and empirical referents. These themes were discussed with the study supervisor, and consensus discussions were held to confirm the themes.

Holistic admission review has increasingly been implemented across health professions education as a strategy to enhance diversity, equity and access to higher education. Evidence indicates that HAR increases the diversity of students who apply for and are admitted to programmes in health professions and schools of nursing (Akintade et al. 2023:24; Aul, Curry & Johnson-Mallard 2022:350; Coplan & Evans 2021:1492; Davis et al. 2020:723; DeCoux Hampton et al. 2022:361; DeWitty 2018:195; Glazer et al. 2016b:306; Jansen et al. 2023:33). Occupational science and occupational therapy departments have implemented holistic selection to ensure equity during the selection process (Anvarizadeh et al. 2023:1; Brotherton et al. 2021:91). Similarly, dental schools have adopted HAR to promote diversity and improve access for underrepresented minority groups (Ardenghi et al. 2024:3; Cunningham & Kiezebrink 2023:505). Holistic admission review has further been incorporated into medical school admissions (Aul et al. 2022:350; Diaz, Huerto & Weiss 2021:223), as well as physical therapy and physician assistant programmes (Brotherton et al. 2021:91). In New Zealand, nursing, pharmacy and health sciences programmes implemented HAR to increase access and equity in higher education (Curtis et al. 2015:1). Some institutions have extended HAR across all incoming programmes to increase institutional diversity and access (Bastedo et al. 2018:783; Black, Cortes & Lincove 2016:337). In the United States, the shift towards HAR was initially influenced by legal developments (Bastedo et al. 2018:785).

The implementation of HAR across the different disciplines shows that the process is not programme specific but rather a systematic admission process. This process is anchored in principles of equity, diversity and inclusion within a specific context. The concept analysis shows that the consistent and multi-disciplinary usage of HAR identifies the process as a developing and valid admission construct.

The implementation of HAR in health professions and nursing entails the assessment of traditional factors such as academic achievement and test scores while simultaneously contextualising these measures and incorporating non-traditional factors such as life experiences, demographic background, leadership, motivation, perseverance and commitment to social justice (Akintade et al. 2023:24; Aul et al. 2022:350; Bruce, Mabizela & Tshabalala 2023:2; Coplan & Evans 2021:1493; Curtis et al. 2015:12; Davis et al. 2020:725; DeCoux Hampton et al. 2022:361; DeWitty 2018:195; Glazer et al. 2016a:307; Jansen et al. 2023:33). Occupational therapy, physical therapy, physician assistant, dental and medical programmes report similar multidimensional criteria (Anvarizadeh et al. 2023:2; Ardenghi et al. 2024:5; Brotherton et al. 2021:92; Diaz et al. 2021:223).

The use of both cognitive and non-cognitive attributes within a specific contextual framework is a definitive and recurring attribute ascribed to HAR. Holistic admission review allows for the evaluation of academic achievement within the applicant’s social, educational and personal context. This is different from traditional selection methods that prioritise academic achievement in isolation.

The assessment of HAR in health professions and nursing is guided by the use of structured rubrics and trained selection committees (Akintade et al. 2023:25; DeCoux Hampton & Apen 2022:345; DeWitty 2018:196; Jansen et al. 2023:33). Nursing programmes in the United Kingdom employ standardised, validated rubrics with explicit criteria (Callwood et al. 2020:2). Occupational therapy programmes emphasise the importance of trained committees and standardised processes to reduce selection bias (Anvarizadeh et al. 2023:3). Although references to standardised rubrics in dental schools vary (Ardenghi et al. 2024:6; Cunningham & Kiezebrink 2023:511), institutions implementing HAR across all programmes highlight the necessity of structured, transparent scoring systems (Holmes & Bear 2022:22).

The use of a standardised rubric implemented by a trained and diverse selection committee is another defining attribute of HAR. The use of the rubric reduces selection bias and increases fairness. By making use of a formal assessment tool, like a rubric, HAR differentiates itself from informal or subjective assessments,thus, enhancing the validity and reliability of the selection.

The HAR selection process in schools of nursing is guided by an explicit philosophy and institutional policy (Coplan & Evans 2021:1493; DeCoux Hampton et al. 2022:362; Jansen et al. 2023:33) that is publicly available to ensure transparency (Akintade et al. 2023:25; Bruce et al. 2023:2). Similar governance structures are reported across health professions (Davis et al. 2020:727; Hossler et al. 2019:835), including medical schools (Diaz et al. 2021:224) and dental schools (Cunningham & Kiezebrink 2023:514).

The requirement for HAR policy and processes to be transparent and publicly available is a further defining attribute of HAR. The combination of the admission mission, vision, philosophy and policy with the admission process enhances accountability and links the selection process with the institutional commitment to diversity and social justice.

Antecedents of HAR entail identifying unjustness in traditional admission processes, unequal access to higher education of underrepresented minority groups, the commitment of institutions to enhancing diversity and in some contexts, legal requirements. These conditions precede and motivate the advocacy of HAR processes.

Holistic admission review results in increased applicant diversity, improved access to higher education for the underrepresented minority groups, increased equity and likely reductions in selection bias. These consequences reinforce HAR as a transformative selection process and not just an alternative selection method.

Results gleaned from the literature entail that the defining attributes of HAR include: (1) context-specific academic evaluation, (2) assessment of both cognitive and non-cognitive factors, (3) use of standardised rubric, (4) trained and diverse selection committee, (5) clear and definite policy and (6) institutional commitment to diversity, equity and social justice. These attributes discern HAR from traditional selection methods and clarify the conceptual boundaries.

### Defining attributes of the concept

The goal of this step was to try to show the attributes that were most associated with the concept and allowed for a broader insight into the concept (Hackett & Ruyak 2022:87; Walker & Avant 2019:173). Criteria are more complex than a limited word definition, they amplify the meaning and suggest direction for the processes of developing empiric knowledge, including theory (Chinn et al. 2022:148). The data were compiled in a table on an Excel spreadsheet with the following headings: Covidence #, Study ID, Title, Author, Date, Country, Antecedent, Defining attributes, Consequences and Empirical referents. The researcher read and re-read the data for the column labelled as defining attributes to determine similar themes that would emerge relating to the defining attributes of HAR of undergraduate students. The common defining attributes were identified, highlighted and grouped together (Walker & Avant 2019:173). The attributes identified during the concept analysis included:

- cognitive, academic, metric, quantitative measures
- non-cognitive, non-academic attributes, qualitative measures
- admission committee, admission lead, faculty, expert-by-experience
- previous experiences
- experience, attributes and metrics (EAM) model
- four core principles of HAR:
  - Selection criteria are broad-based and linked to the school’s mission and goals, and promote diversity.
  - Balanced EAM to assess applicants with the intent to create a diverse selection pool, with data grounded in evidence support.
  - Admission committee give individualised consideration to how each applicant may contribute to the school environment and profession, weighing and balancing the criteria to achieve outcomes.
  - Race and ethnicity may be considered as factors when making admission-related decisions (where permitted by law) only when consideration is tailored to achieve mission-related education interests and goals associated with diversity (Morrow 2021:257).

### Identifying a model case

A model case or exemplar case is described as a depiction of a situation, experience or event that is the best representation of the concept according to the researcher’s understanding (Chinn et al. 2022:144). In other words, a model case is an example of the use of the concept that demonstrates all the defining attributes of the concept being analysed (Hackett & Ruyak 2022:88; Walker & Avant 2019:174). The model case was based on the ideal selection process of undergraduate students implemented at a nursing education institution (NEI). The scenario addressed all the defining attributes identified during the concept analysis, which included a selection process guided by policy addressing the four core principles of HAR. The use of both cognitive and non-cognitive measures is implemented. The selection was done by a diverse, trained selection committee using a standardised rubric.

### Identifying additional cases

By identifying other cases that are not exactly the same as the concept of interest but are similar to it or contrary to it, the researcher will be able to make better judgements about which defining attributes or characteristics have the best fit (Walker & Avant 2019:175). The basic purpose of the additional cases is to assist in the decision-making process of which defining attribute is of interest and which ones are not (Walker & Avant 2019:175).

#### Identifying a borderline case

Borderline cases are found when the same word is used in a different context (Chinn et al. 2022:147). It also entails that this example uses most of the defining attributes associated with the concept, but not all of them (Walker & Avant 2019:175). Borderline cases seem to be inconsistent in some way (Walker & Avant 2019:175). Borderline cases include using both qualitative and quantitative measures but with no measures of reliability or validity.

#### Identifying a contrary case

Contrary cases are the antithesis of the model case, those cases that certainly do not exemplify the concept (Chinn et al. 2022:146; Walker & Avant 2019:177). During the process of developing a contrary case, the researcher should ask: What makes this instance different from the concept? (Chinn et al. 2022:147). Contrary cases are helpful as it is often easier to say what something is not, rather than what it is, and that clearly excludes certain attributes (Walker & Avant 2019:177). Contrary cases include using either qualitative or quantitative selection processes.

#### Identifying a related case

Related cases are those cases that represent a different but similar concept (Chinn et al. 2022:147; Walker & Avant 2019:176). The concepts are like the concept being analysed and thus connected in some way (Walker & Avant 2019:176). These cases share several criteria with the concept, but one or more criteria will be particularly associated with the model case and will distinguish the exemplar case from the related case (Chinn et al. 2022:147). Related cases allow for close examination of the concept that assists in clarifying what is deemed as a defining attribute and what is not (Walker & Avant 2019:176). Related cases include cases where holistic admission processes are implemented in faculties other than nursing.

### Identifying antecedents and consequences

Antecedents are those events or incidents that must occur or be in place before the concept can occur (Hackett & Ruyak 2022:88; Rodgers 1989:334; Walker & Avant 2019:178). Antecedents in HAR within the context of nursing were identified as diversity and/or equity, success, values and/or qualities of the student, reliability and validity and transparency and bias and quality of care or patient outcomes or needs or satisfaction (Zerwic et al. 2018:417). Consequences of HAR within the context of nursing were identified as metrics related to increased bias, decreasing the applicant pool and reducing bias (Diaz et al. 2021:223), qualitative measures alone not enough and need to be combined with quantitative measures (Crawford et al. 2021:2511; Gale et al. 2016:125; Jeffrey, Harris & Sherman 2019:70; Joubert, Downing & Kearns 2022:7; Liu 2022:14; Murray & Noone 2022:141; Roach et al. 2019:127; Thomsen 2018:347; Zerwic et al. 2018:420) and the biggest challenge is related to inter-rater reliability, validity and transparency of qualitative measures (Griffin et al. 2018:1; Katz & Vinker 2014:2; Macduff, Stephen & Taylor 2016:45; Rideout 2017:56; Vierula et al. 2020:129). As such, there is a need for a rubric, tools and/or policies and procedures to facilitate the implementation of HAR. These tools should be evidence based (Cunningham & Kiezebrink 2023:511). Finally, HAR increases diversity, which in turn increases student success and leads to a positive learning environment (Coplan & Evans 2021:1510; Grabowski 2016:4) through increased community involvement, improved student collaboration and communication and a greater acceptance of opinions that differed from their own (Breland 2020:23; Glazer et al. 2016a:307).

### Defining empirical referents

The following aspects were identified as empirical referents of HAR:

- Multiple mini-interview (MMI)/interviews.
- Standardised tests/Grade Point Average (GPA)/aptitude tests.
- Recommendation letters and personal statements.
- Essays (reflective, admission or writing assignments).
- Emotional intelligence scales and empathy.
- Sedlack non-cognitive questionnaires.
- Other questionnaires: Situational Judgement Test (SJT), Casper, Personality, Psychometric, Seldman’s retention, Swall model, Sternberg’s, Self-directed search (SDS) questionnaire, Student Readiness Inventory (SRI).

## Discussion

The results of HAR will be discussed as they appear in Table 1.

**TABLE 1:** Results of holistic admission review.

Category (Walker & Avant 2019)	Defining attributes and related connotations
Antecedent	Increased diversity of the population. Increased need for student success – Academically and professionally.
Process and /or procedure	Triggered by: Lack of workforce diversity because of decreased student body diversity. Bias and reduced reliability, validity and transparency during the selection process. Phase 1: The NEI to develop policies, procedures, mission and vision to address HAR. Four core principles of HAR. Phase 2: Identifying all the qualitative measures required as part of the HAR. Phase 3: Identifying all the quantitative measures required as part of the HAR. Phase 4: Implement measures to ensure reliability and validity of the selection process. Standardised selection process Selection committee Training Standardised rubric
Outcome	Promoting a diverse and inclusive student body, enhancing transformation and growth. Delivering superior quality of care to patients and elevating patient well-being. Empowering students for success, boosting retention and shaping future leaders.

*Source*: Walker, L. & Avant, K., 2019, *Strategies for theory construction in nursing*, 6th edn., Pearson Education Inc., New York, NY

HAR, holistic admission review; NEI, nursing education institution.

### Antecedent

A thorough search of the literature was conducted in order to extract the relevant uses, attributes, essence and outcomes of HAR in student selection. The exploration uncovered antecedents of HAR within the context of student selection, as an increased diversity of the population and an increased need for student success, both academically and professionally.

Populations are changing. It is becoming diverse across racial, ethnic and cultural groups (Zimnicki et al. 2022:375). With people emigrating across countries and borders, we are seeing different cultures living and working together, and as such, they will seek medical care together. It has been found that minority populations, i.e. in terms of race, ethnicity and gender, experience worse health outcomes than majority populations (Rosenberg 2019:669; Stratton & Elam 2014:2). There is overwhelming evidence that indicates a lack of diversity increases health disparities in underrepresented groups (Akintade et al. 2023:21; Ardenghi et al. 2024:1; Joubert et al. 2022:6; Langer et al. 2020:230; Mthimunye & Daniels 2019:61; Rideout 2018:3; Roach et al. 2019:125). These disparities are associated with patients not feeling comfortable to seek advice or even follow the advice of someone they do not trust. Diversity has been recognised as an important factor in improving health outcomes for patients (Jansen et al. 2023:32; Roach et al. 2019:127; Rosenberg 2019:669). This diversity is gained through gender, racial and ethnic diversity (Al-Alawi, Oliver & Donaldson 2020:2; Davis et al. 2020:726; DeCoux Hampton et al. 2021:2, 2022:362; Moche, De Swardt & Havenga 2021:13; Morrow 2021:258; Sommers et al. 2022:341; Wros & Noone 2018:211; Zimnicki et al. 2022:377). This is important as healthcare providers are the ones who engage with patients the most frequently and intimately (Akintade et al. 2023:22).

The health needs of the population are changing and becoming increasingly complex, leading to a greater demand for registered nurses (Al-Alawi et al. 2020:1). The calls for greater efficiency in higher education are increasing (Black et al. 2016:336). Provider diversity is associated with better educational experiences for health professions students (Rosenberg 2019:669). Holistic admission review improved student success, which was measured by academic performance and retention (Anvarizadeh et al. 2023:2; Grabowski 2016:41; Rosenberg 2019:669; Schreurs et al. 2018:2; Twidwell & Records 2017:11). Academic success is linked to student retention and completion of the academic programme (Akintade et al. 2023:25; Aul et al. 2022:351; Wambuguh, Eckfield & Van Hofwegen 2016:93). A method of enhancing success is through the implementation of a diverse and inclusive environment (Akintade et al. 2023:25) and thus increasing the student experience (Akintade et al. 2023:26). In selecting the right student, nursing workforce performance will improve in the future and ultimately will improve the safety and well-being of patients (Bruce et al. 2023:2; Lancia et al. 2018:115; Stenhouse et al. 2016:1; Zamanzadeh et al. 2020:2).

### Process

The process of HAR involves four phases. The phases are steps to be followed to develop and implement HAR. There are two main triggers relating to the commencement of this phase. Firstly, the trigger relates to a lack of workforce diversity as a result of decreased student body diversity. The lack of workforce diversity is directly linked to the lack of diversity in the educational programmes (Anvarizadeh et al. 2023:2). This lack of representation is largely because of decreased access to higher education (DeCoux Hampton et al. 2022:2). Longstanding selection structures prioritise the dominant gender, racial and ethnic groups (Ardenghi et al. 2024:1; Brotherton et al. 2021:91; Okorie-Awé et al. 2015:1107). The only way to diversify the healthcare workforce is for the institutions to diversify the student body (Akintade et al. 2023:22). For an admissions programme to be successful, it should increase diversity as well as improve access for students who previously would not have had access (Boske et al. 2018:4; Holmes & Bear 2022:19; Sandlin 2019:7).

Secondly, the trigger refers to bias and reduced reliability, validity and transparency related to the selection process (Rideout 2018:3; Van der Merwe et al. 2016:76). Selection processes traditionally have been known to introduce bias. Making use of a single variable, especially metrics only, introduces bias as not all students have had equal access and opportunity (Anvarizadeh et al. 2023:3). Non-cognitive variables used by themselves have been found to have low validity and reliability (Ardenghi et al. 2024:6; Hossler et al. 2019:841; Nicola, Savitz-Romer & DiLorenzo 2023:102). Holistic admission review has been adopted as the standard to address bias associated with over-reliance on standardised test scores (Bastedo et al. 2018:785; Lancia et al. 2018:119).

Phase 1 of the HAR process requires the NEI to develop policies, procedures, mission and vision to address HAR (Gay et al. 2018:152; Nicola et al. 2023:106). This process of development is to be guided by the four core principles of HAR. An admission statement of philosophy is needed to guide the development of the process and rubric to evaluate applications (Akintade et al. 2023:24; Verity 2022:75). The HAR initiative needs to be mission-driven, with language and activities embedded throughout the institution that reflect diversity as an important element (Ardenghi et al. 2024:6; Rosenberg 2019:670). This will include the model to be implemented, for example, the EAM model, the recruitment of applicants, the selection process itself, how all measures will be assessed and the use of rubrics or the development of such rubrics (DeCoux Hampton & Apen 2022:344; DeWitty 2018:196; Lancaster, Baseman & Smolinski 2020:260; Mann et al. 2022:43; Manske, Johnson & Brown 2023:556; Maude & Kirby 2022:77; Ober et al. 2023:58; Rosenberg 2019:670; Sommers & Wirawan 2019:2). A school’s objectives and mission will dictate the who and how of recruitment (Holmes & Bear 2022:23; Kridiotis, Bezuidenhout & Raubenheimer 2016:208; Rosenberg 2019:670). For the policies to be successful, the institution would need stakeholder or faculty buy-in (Rosenberg 2019:670).

Phase 2 refers to the NEI identifying the qualitative measures that need to be assessed during the selection process. This will be guided by the policies developed in Phase 1. There needs to be a common, agreed-upon understanding of which variables are important (Kennedy et al. 2020:1050; Matthews et al. 2022:98; Murray & Noone 2022:141; Niessen & Meijer 2017:436; Rosenberg 2019:670). These can be referred to as the Experiences and Attributes of the EAM model (Anvarizadeh et al. 2023:3; Rosenberg 2019:670). Identify the attributes and map these qualities, for example, psychometric inventories (Hossler et al. 2019:841; Klingenberg & Pelletier 2019:313; Mabope et al. 2022:69) and emotional intelligence (Rankin 2013:2718) to the application criteria (Anvarizadeh et al. 2023:3; Coplan & Evans 2021:1493; Okorie-Awé et al. 2015:1107; Schreurs et al. 2018:1). Values deemed as important include trust, integrity, accountability, personal development, person centred, teamwork and caring (Griffin et al. 2018:3; Rankin 2013:2718; Snowden et al. 2018:437; Stenhouse et al. 2016:2; Traynor et al. 2017:1445; Watson et al. 2022:1). Finally, to identify the weighting of the non-cognitive measures (Anvarizadeh et al. 2023:3).

Phase 3 is similar to Phase 2, except that in this step, the quantitative measures are identified. Phases 2 and 3 are interchangeable; however, they can only occur once the policies have been developed in Phase 1. There needs to be an understanding of which merits are deemed to be important, and these can be referred to as the Metrics of the EAM model (Curtis et al. 2015:2; Diaz et al. 2021:224; Gale et al. 2016:125; Joubert et al. 2022:6; Kennedy et al. 2020:1050; Langer et al. 2020:230; Macduff et al. 2016:42; Rosenberg 2019:670; Twidwell & Records 2017:1). Establishing a metric floor would be essential to academic success, which would refer to moving away from metrics as the most important measure to a more balanced consideration of metrics, experiences and attributes (Rosenberg 2019:670). Identify the cognitive measures and determine the weighting of these measures without overweighting the cognitive measures (Anvarizadeh et al. 2023:3; Mabope et al. 2022:69; Verity 2022:13).

Phase 4 refers to the measures that will be implemented to ensure the reliability and validity of the selection process. These measures include standardising the selection process (Jeffrey et al. 2019:65; Nicola et al. 2023:105; Roach et al. 2019:127), the establishment of a selection committee (Kennedy et al. 2020:1051; Thomsen 2018:335; Watson et al. 2022:6), specific training for this selection committee as well as the development of a standardised evaluation rubric (Kennedy et al. 2020:1051; Mann et al. 2022:46; Murray et al. 2022:127; Tetley et al. 2016:155).

### Outcome

Holistic admission review is a way to select a student by considering the student as a whole and not just simply academic merit. The selection of a student who can perform academically, complete the programme, enter the workforce and be a valuable member of the team is vital to improving the quality of health of a population (Macduff et al. 2016:42; Okorie-Awé et al. 2015:1107). Holistic admission review is a method to transform selection, which is biased and limited to transparent, valid and inclusive (Coplan & Evans 2021:1493). Firstly, from the concept analysis, the outcome of HAR is to promote a diverse and inclusive student body that is able to enhance transformation and growth (Murray & Noone 2022:141; Niessen & Meijer 2017:437). Secondly, HAR allows for selecting students who are able to deliver a superior quality of care to patients and are able to elevate patient well-being. Thirdly, HAR empowers students for success, boosting retention and shaping future leaders (Aul et al. 2022:351; Mabope et al. 2022:69; Snowden et al. 2018:434).

### Theoretical definition of the concept holistic admission review in nursing education

Concept analysis provided a theoretical definition as it allowed for an examination of the ways in which the concept of HAR was used in existing writings (Chinn et al. 2022:143). Thus, a theoretical definition delineates how a concept is and should be in all instances. In this study, a theoretical definition was formulated from the antecedents, defining attributes and their related connotations in the context of undergraduate student selection. Thus, the theoretical definition for HAR based on the concept analysis is that HAR is a systematic process guided by the four core principles of HAR, which informs the nursing education institution’s mission, vision and selection policies. HAR is triggered by a need to select a diverse student body, which in turn would diversify the workforce, to address the health needs of a diverse population and improve the access and quality of care. The process involves the assessment of both qualitative and quantitative measures in an unbiased way to select the ideal student who would be successful both academically and professionally.

### Theoretical validity

Theoretical validity refers to the extent to which the interpretation of the results from the concept analysis aligns with the theoretical definition (Tsimane 2018:85). The validation process necessitates various methods to assess whether the explanation accurately reflects the definition (Chinn et al. 2022:11). Theoretical validity was ensured using four principles of philosophical perspectives, namely: the epistemological, pragmatic, linguistic and logical philosophies (Tsimane 2018:85). The examination and description of definitions and uses of HAR from the literature ensured the theoretical definition.

### Strengths and limitations

The exploration of the meaning of HAR in nursing education, as well as the development of a theoretical definition of HAR in nursing education, is new. The analysis will contribute to the development of a model that could facilitate the implementation of HAR in nursing education in South Africa. Only articles published in English and Afrikaans were included. Studies published in other languages were excluded, and could lead to missing valuable data. The researcher also excluded the use of HAR in postgraduate student selection, which could lead to biased selection.

### Relevance for clinical practice

This concept analysis will refine the research questions for exploring HAR’s meaning in nursing education in South Africa. This is achieved by identifying key attributes of HAR, paving the way for its integration into South African nursing education. Through this process, the development of a model for the implementation of HAR in nursing education in South Africa will be developed to facilitate equitable student selection in South Africa.

## Conclusion

The use of Walker and Avant’s eight-step method of concept analysis allowed for the exploration and description of the meaning of HAR within the context of nursing education. The results identified three categories, namely antecedents, process and outcomes, as depicted in Table 1. The results add new knowledge to the selection processes of nursing students and will be used for further research. This includes the exploration of HAR within the context of nursing education in South Africa, as well as the development of a model for the implementation of HAR for student nurses within the context of South Africa.
